# Enhanced Approach Using Reduced SBTFD Features and Modified Individual Behavior Estimation for Crowd Condition Prediction

**DOI:** 10.3390/e21050487

**Published:** 2019-05-13

**Authors:** Fatai Idowu Sadiq, Ali Selamat, Roliana Ibrahim, Ondrej Krejcar

**Affiliations:** 1 Faculty of Engineering, School of Computing, UTM & Media and Games Center of Excellence (MagicX), Universiti Teknologi Malaysia, 81310 Johor Bahru, Malaysia; 2 Faculty of Physical Sciences, Ambrose Alli University, P.M.B 14, 310101 Ekpoma, Edo State, Nigeria; 3 Center for Basic and Applied Research, Faculty of Informatics and Management, University of Hradec Kralove, Rokitanskeho 62, 500 03 Hradec Kralove, Czech Republic; 4 Malaysia Japan International Institute of Technology (MJIIT), Universiti Teknologi Malaysia, 54100 Kuala Lumpur, Malaysia

**Keywords:** context-aware framework, accuracy, false negative rate, individual behavior estimation, statistical-based time-frequency domain and crowd condition

## Abstract

Sensor technology provides the real-time monitoring of data in several scenarios that contribute to the improved security of life and property. Crowd condition monitoring is an area that has benefited from this. The basic context-aware framework (BCF) uses activity recognition based on emerging intelligent technology and is among the best that has been proposed for this purpose. However, accuracy is low, and the false negative rate (FNR) remains high. Thus, the need for an enhanced framework that offers reduced FNR and higher accuracy becomes necessary. This article reports our work on the development of an enhanced context-aware framework (EHCAF) using smartphone participatory sensing for crowd monitoring, dimensionality reduction of statistical-based time-frequency domain (SBTFD) features, and enhanced individual behavior estimation (IBE_enhcaf_). The experimental results achieved 99.1% accuracy and an FNR of 2.8%, showing a clear improvement over the 92.0% accuracy, and an FNR of 31.3% of the BCF.

## 1. Introduction

Crowd abnormality monitor (CAM) is a process of determining individual behavior in a crowd to prevent accidents in crowd-prone areas. Crowd monitoring using activity recognition (AR) to analyze individual behavior is maturing rapidly due to the current advancement in sensor technologies [1]. Increased research focus on human activity recognition (HAR) in diverse application domains highlights the significance of human–computer interaction (HCI) [2]. Two conventional methods are employed in the analysis of abnormal behavior in crowds. According to Zhang et al. [3], the “object-based” method identifies a crowd as a collection of individuals, while segmentation methods are used for analyses of crowd behaviors. In crowd behavior analysis, the performance of segmentation or detection of objects is usually faced with the complexity in the detection of objects [3]. Previous studies have demonstrated the object-based method with individual activity recognition. Issues in ongoing research have been extensively discussed, with initial solutions suggested in [4]. Context-aware approaches have been proposed previously; for example, [5]. However, only one [6] focused on crowd abnormality monitor and mitigation with the use of individual AR. However, the threshold used for crowd density in terms of the prediction of crowd condition is unclear [6]. An efficient approach should be able to accurately determine the number of persons within a square meter in order to prevent accidents during an emergency in a crowd scenario [7]. In the study by [6], the simulation was done inside a university building and conducted with a system of CAM [6], thus reducing the practical applicability of the system. Therefore, an alternative with high accuracy performance and a low false negative rate (FNR), which measures the false alarm to promote the efficient and reliable prediction of crowd conditions based on individual behavior [6], is needed. This will be based on an extension of the proposed basic context-aware framework (BCF) proposed [6]. A potential solution is to advance the previous BCF using the reduction of relevant statistical-based time-frequency domain (SBTFD) features with improved accuracy, reduced the FNR, and IBE_enhcaf_ for individual and crowd condition prediction.

The motivation of this article proposes an enhanced context-aware framework using IBE_enhcaf_ to improve the safety of human lives in a crowd-prone environment. The proposed approach utilized reduced features, with high-accuracy performance previously reported [4,8]. This study reports the result of an ongoing study on other sensor data validation, which included the effect of low FNR, and a clear definition of crowd density threshold for individuals per square meter (m^2^) for crowd monitoring. The proposed approach employs the crowd density definition suggested in [7] and utilizes individual contexts from sensor signals in real time. In addition, the detection of five or more persons per m^2^ is considered an extremely high density [9] to minimize the risk of accident in a moving crowd. The suggested solution promises accurate and reliable feedback to likely accident victims in an unforeseen situation. In this article, the context-aware framework is defined as a BCF that utilizes contexts such as individual user activities, location, and time [6]. The contexts are hidden information derived from smartphone sensor data [6]. The contributions of this article are:

(1) To present the validation result of other sensors used for individual behavior estimation (IBE) to extend the BCF. 

(2) To suggest a clear crowd density threshold (CDT) per m^2^ using a low FNR from reduced features to extend BCF. 

(3) To propose an enhanced approach with reduced SBTFD features and modified IBE for crowd condition prediction with CDT to improve on BCF.

The proposed solution has the potential to minimize incessant death occurrences in social gatherings through a viable technology concept. The rest of the article is organized as follows: Section 2 discusses the current approaches to crowd monitoring, Section 3 presents the materials and methodology used in the study, Section 4 presents experimental results for the investigated issue to achieve the contributions in the article. The results are discussed in Section 5, while Section 6 addresses the conclusion and future work.

## 2. Current Approaches in Crowd Monitoring System

The crowd monitoring system (CMS) currently has three approaches, namely: (i) computer vision-based methods, (ii) sensor data analysis, and (iii) social media data analysis [10]. The most commonly used is sensor data analysis, which is also employed in this study [11] for several reasons. These include (i) a tendency for the provision of accurate and real-time information, (ii) nowadays, the new sensors on smartphones having the potential to revolutionize how we manage information, (iii) offering safety and enhancing security if well utilized in crowded places, (iv) wider coverage, as smartphones are used by almost everyone, and (v) feedback to potential victims in case of accidents [12]. Besides, sensor data analysis is widely used in AR with promising results [1,2,5]. Several feature extraction methods (FEM) have been employed in recent studies [13,14]. Table 1 presents the strengths and limitations of existing feature extraction methods.

The following section presents an analysis of FEM, including time domain (TD), frequency domain (FD), and feature reduction, and highlights those that can potentially be used for individual and crowd condition monitoring. Then, feature reduction based on feature selection methods (FSM) is examined for CMS for the minimization of time, classification, and accurate prediction. Related studies in context-aware frameworks are also discussed. 

### 2.1. Time Domain (TD)

TD features include mean, median, range, variance, maximum, minimum, skewness, and kurtosis, to name a few. The features are widely used in HAR [15,16,17]. According to [17], the integral method has been applied to extract energy expenditure information from raw sensor signal data, where the total integral of the modulus of acceleration (IMA) was employed. The method is referred to as the time integral of the module of accelerometer signals, and is expressed in Equation (1):

(1) $$IMA_{tot}=\int_{t=1}^{N}\left|a_{x}\right|dt+\int_{t=0}^{N}\left|a_{y}\right|dt+\int_{t=0}^{N}\left|a_{z}\right|dt$$

where $a_{x},a_{y},a_{z}$ represent the orthogonal components of acceleration, t denotes time, and *N* is the window length. Some of the methods of extracting features rely on the ability to transform input signals to and from different domains [14]. To apply feature computations on a smartphone, one needs to be careful due to computational complexity as a result of limited memory, processing time, and battery lifetime. According to [18], almost all TD features are suitable for mobile devices, because their correlation operations have higher computational cost. A feature extracted from the raw sensor signal’s data from individual activity recognition is such a piece of information, and can be used when classifying activity recognition to determine the characteristics of the individual in a crowd scenario in this thesis. In order to create features from the AR sensor raw dataset, different methods and mathematical calculations are applied to the raw dataset, and new features are extracted. Other time domain features such as zero crossing, signal vector magnitude, the signal magnitude area, and angular velocity have also been used in AR [19,20].

### 2.2. Frequency Domain (FD)

Features in this domain are important because the Fourier domain in AR sensor data has a much greater range than the AR in the spatial domain. To be sufficiently accurate, its values are usually calculated and in float values. Fast Fourier transform (FFT) also preserves information from the original raw signal and ensures that important features are not lost as a result of FFT [21]. FD splits the signal into sinusoidal waves with various frequencies using Equation (2):

(2) $$f=\int_{1}^{w}x(t)e^{-j2\pi ft}dt;\;x(t)=\int_{1}^{w}X(f)e^{j2\pi ft}dt$$

where *t* = time; *f* = frequency; *X(f)* = inverse Fourier transform; and *x(t)* depicts Fourier transformation [22].

The proper selection of FD feature and sampling frequency is a key factor for extracting the frequency components; an inability to realize this may result in a false prediction of an individual in a crowd [3]. Zheng [3] transforms *x(t)* to overcomes the drawback of inaccurate detection by introducing a frequency domain component and obtaining relevant information for AR [3,23]. Other important domains include the wavelet domain (WD), which are better noted in the analysis if irregular data patterns are used; that is, impulses exist at different time intervals [12], and therefore, require the selection of a proper mother wavelet. The heuristic domain (HD) works by using the assignment of the correct value to suggest the best corrective measure of sensor signals [16]. Therefore, HD requires input from multiple experts aggregates the result. The time domain–frequency domain (TDFD) produces an efficient performance for individual’s representation in the crowd [14]; however, the use of FFT_RMS as the only FD may not assume the performance of other TD features. 

Table 2 presents a synthesis of existing FEMs and their names in AR. It shows the features used in a crowd condition, the application domain, and the researcher, and those that have not been used in crowd conditions are also indicated. Table 2 shows that only conventional FEMs have been used in previous crowd-related research with Mean, Std, along x, y, and z [16,18,22], and variance along x, y, and z [14,18]. This could be responsible for the observed inaccuracy of 92% reported for CAM, which has also been noted by [24] to be generally low. It can also be noted that some salient TDFD features that are capable of accurate prediction were overlooked in the BCF, thus strengthening the need for further studies. 

### 2.3. Related Works on Feature Reduction, Context-Aware Framework (CAF), and Activity Recognition (AR) 

Feature reduction methods are important approaches that help avoid the cause of dimensionality [30], that is, the number of feature spaces in a feature vector. It targets a reduction in the number of previously used features on a mobile device in AR. High dimensionality on the accuracy of classification performance has been an important domain of research in HAR [31,32]. Feature reduction can facilitate the early detection of an emergency in an unforeseen circumstance [29]. Thus, the risk associated with individual activity recognition (IAR) in a crowd condition can be minimized by the reduction of FNR. The issue of high false alarm with FNR was not addressed in BCF. The solution proposed in our previous work as Phase 2 was reported [4].

The review of AR recognition works on individuals and crowds explains the potential of features dimensionality reduction for accurate and efficient crowd conditions; however, a feature reduction-based feature selection method has never been applied for this purpose. The work of [33] on early recognition supports this objective; it predicts a one-shot learning-based pattern transition for early detection recognition. A great benefit of the approach proposed by [34] utilized a smaller number of features for the prediction of ovarian cancer survival, and achieved very limited computational efforts. The use of a smart selection of a lesser number of relevant features compared with the number of features used with FEM in BCF diminishes the computational effort greatly, and reduced the false negative alarm. Moreover, an unclear definition of CDT has been noted by [7,9] as a major challenge in BCF. An inappropriate threshold of high density used for individual behavior estimation by [6], and a lack of feedback to victims resulting to a high false alarm in an emergency led to an unreliable prediction of crowd conditions, such as for example crowd abnormality behavior. Chang et al. [35] introduced a context-aware mobile platform for an intellectual disaster alerts system (IDAS); it focused on how environmental changes can result in accidents and disasters. According to the authors, a quick and accurate alert delivered to victims is essential in a disaster situation. However, their work focuses on addressing disaster issues, rather than crowd monitoring for safety.

Context-aware computing, an application concept that can sense the physical environment and reacts accordingly, was proposed by [36]. It is aimed at facilitating the quick and efficient development of a framework that combines context-aware service and machine learning [36]. The study led to the development of context-aware and pattern oriented machine-learning framework (CAPOMF). It focused on how commuters can avoid potholes to save vehicle repair costs. In previous context-awareness research, machine learning is rarely used [36,37,38] for the realization of context-aware framework. The studies of [6,39] also emphasized that context-aware application and its services remain open research issues. Prior to [6], no context-aware research with activity recognition have been applied or proposed for crowd abnormality mitigation in the literature. The outstanding problems that constitute a challenge in context-aware research regarding their affects on crowd disaster mitigation are itemized as follows:

(1) Context acquisition, modeling, inference, and sensing.
(2) Determination of appropriate sensors and the nature of contexts to be acquired.
(3) Real-time management of sensors and context-based action generation.

As of June 2018, context-aware computing was worth US$120 billion [40]. Its research finds application in many domains with only few in disaster management. The extant literature highlights three methods used in context-aware framework: (i) scenario-based with a hypothetical example using a develop application, (ii) comparative analysis using a side-by-side comparison of components [41], and metric evaluation with accuracy, precision, recall, and f-score with an experiment on related activities [35]. Table 3 presents related works and highlights gaps in previous research.

## 3. Materials and Methods 

This section presents the methodology employed in this study. It provides a description of the development of the context-aware activity recognition application used for data collection, data validation outcome, adopted and modified algorithm implementation, and results in analysis approaches.

**Figure 1:**
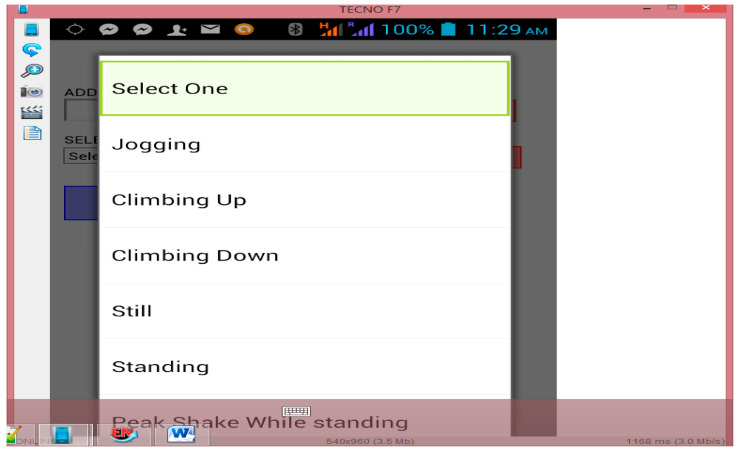
Sensor signals dataset collection interface used by volunteers during the experiment.

We developed an Android application called Context Activity Data Collector (CADC) based on Java programming as a client, and the crowd controller station (CCS) as a server to store the CADC in real-time for offline data analysis. The CADC runs on an Android 3.0.2 version of a Samsung Galaxy SM-G530H. Figure 1 shows the CADC data collection interface. An example of the sensor signals collected at a Malaysian public institution between March and April (2015) is shown in Figure 1. The eight (8) classes considered in the experiment conducted are selected from multiple possible conditions of an individual in the considered scenario. The scenarios considered are: climb down (V1), climb up (V2), fall (V3), jogging (V4), peak shake while standing (V5), standing (V6), still (V7), and walking (V8). 

Several instances were captured for each scenario performed by volunteers (node S), yielding 22,350 class instances. In this case, S is referred to as the volunteers that make use of Figure 1 in the experiment conducted. The class instances obtained from S during the experiment include V1: 1975, V2: 2410, V3: 3159, V4: 2952, V5: 2937, V6: 2757, V7:3230, and V8: 3470 for dataset D1. The validated results of other sensor signals (captured as six additional classes, V12 to V18) for D1, which include a digital compass, longitude, latitude, and timestamps used for individual behavior estimation, were reported for dataset D1 based on IAR. Table 4 summarized the D1 dataset used for this research. 

### 3.1. Methodology for the Proposed Enhanced Approach

**Figure 2:**
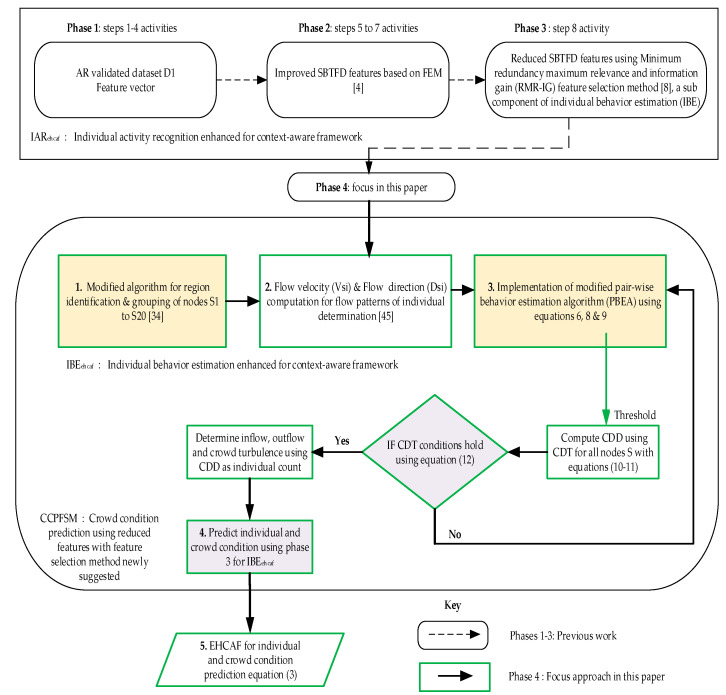
The process flow of the methodology used for the enhanced context-aware framework approach (EHCAF).

The methodology in this article focuses on Phase 4 of Figure 2, while phases 1–3 were activities presented in the previous work [4,8]. They are important to achieve Phase 4 focused in this article as stated in the objective highlighted in Section 1, and the need for the reflection of these parts in Figure 2 for clear flow and understanding of this article. 

A high accuracy and reduction of a negative false alarm are highly desirable and central to crowd condition prediction; however, the approach cannot be adopted without adequate changes to the algorithm using the same data collection with the activity recognition method as shown in Figure 1 using Table 4. This was done by adopting the suitable threshold, which is called the crowd density threshold (CDT) (Figure 2) in Equation (4), while modifying the algorithms presented in BCF with a clear threshold definition of crowd density estimation to accurately detect individual per m^2^ in crowd scenarios experimented. The crowd density in this study is defined as >2 persons/m^2^. In order to achieve the stated objectives, the following tasks were carried out as summarized in Figure 2:

Step 1: Design: experimental; data type: sensor-based real-time IAR; Sample: 20 volunteers; provided: 22350 instances for D1 dataset.

Step 2: Procedure: development of CADC application (Figure 1) with algorithm implemented based on CDT using Java installed on volunteers’ phones; sensors (digital compass, longitude, latitude as Global Positioning System (GPS) data for location etc., as presented in Table 4. 

Step 3: Functioning of CADC: internet-enabled with hotspots; 50 to 100 m^2^ coverage.

Step 4: Server setup: crowd controller station (CCS); volunteers (node S) launch the CADC app by pressing the start button; select activity scenario; perform each for 10 min while maintaining a range of 1 m^2^ to each other, which was done collectively until all activity is reached; CCS store the sensor signals’ collected data in text format; each volunteer stops the app as specified to end the data collection; duration was 5 h for each round of data collection. The guideline in the previous AR data set is employed [11,13,20]. The D1 collection became necessary because the sensors required were not available in the public domain [11,13,20] at the time of this study. 

Step 5: Validation: The validation of raw sensor signals [44] was performed using an analysis of variance (ANOVA). This helps for the significant test of the dataset used in this study. 

Step 6: Data analysis: Missing data was handled by employing moving average; noise removal from D1 was achieved using segmentation with 50% overlapping based on 256 sliding windows; for detail, see [4]. 

Step 7: Improved SBTFD features with newly suggested 39 features based on FEM (total 54 features) yields 7.1% accuracy improvement; this was implemented in Python; and reported in [4]. 

Step 8: Feature reduction using a feature selection method newly introduced to this domain produced seven (7) effective features; this again yields 99.1% accuracy, which is also an enhancement in AR and crowd monitoring studies; details are provided in [8]. 

This section described the procedure for enhanced IBE. Following the AR in steps 7 and 8; it is necessary to obtain other necessary features that can identify and estimate the behavior of an individual [6]. It begins with the implementation of a modified algorithm for the identification and grouping of individual participants (smartphone) as node S by the crowd controller station (CCS) using GPS as sensor data [5]. This is followed by the implementation of adopted algorithms, which determines abnormal movement behavior among individuals using the flow velocity Vsi estimation and flow direction Dsi identification [44]. The Vsi and Dsi were computed using the sensor fusion method based on Kalman filter as reported in [44]. 

The next stage picks the Vsi and Dsi, and combines them with the seven best (reduced) features previously achieved in step 8 from each class of activity scenario e.g., V2; for detail, see [33]. Thereafter, the combined Vsi, Dsi, and reduced features were used as input to modify the pairwise behavior estimation algorithm (PBEA). The PBEA was implemented to identify and determine the behavior of the individual in a crowd with a disparity value computed using the disparity matrix. The final stage employs the IBE using the reduced features based on CDT to evaluate the individual crowd density determination (CDD) per m^2^. The CDD help to appraise the inflow and outflow of moving individuals to ascertain crowd turbulence. This was realized using the CCS, which triggers up a context-aware alert to predict the abnormal behavior of an individual and crowd condition. It also determines the participation of the individual in a crowd scenario based on disparity values to develop the proposed approach, an enhanced context-aware framework (EHCAF), which is an improvement on the BCF. 

The following sections present details of the steps in the research methodology after the IAR using the reduced features in Phase 3 to achieve an IAR flow pattern. The flow pattern differentiates the behavior of one node from the other nodes in the experiment [5]. In the following section, a brief description of these sensors’ validation is presented. 

### 3.2. D1 Validation of Sensor Signals apart from Accelerometer Data

The result of the accelerometer signals of D1 was earlier reported [4]. D1 validation was carried out to validate the processed raw sensor signals for other sensors used for IBE_ehcaf_ in this article. The validation task was carried out to ascertain the quality of the D1 dataset displayed in Figure 1. We have applied the statistical validation technique (SVT) commonly used in the literature [3,22] based on the parametric nature of the dataset. For the validation, two hypotheses were formulated and tested using IBM SPSS 22.0. The hypotheses are as follows:

(1) Null hypothesis H_0_: $\mu_{1}=\mu_{2}=\mu_{3}\ldots ,\mu_{11}$; there is no significant difference between the means of the variables V12, V13, …, V18 used for the analysis of D1 for prediction in this study.

(2) Alternative hypothesis H_A_: µ_1_ ≠ µ_2_ ≠ µ_3_ ≠…; there is a significant difference in at least one of the means of the variables V12, V13, …, V18 used for the analysis of D1 for prediction in this study.

#### 3.2.1. Reduced Features from Improved Statistical-Based Time-Frequency Domain (SBTFD) 

This section discusses the reduced features from SBTFD employed for an enhanced context-aware framework for individual activity recognition (IAR_ehcaf_) in (Phase 2 of Figure 2) based on improved SBTFD features reported in our previous works [4]. In this article, we focus on the individual behavior estimation enhancement (*IBE_ehcaf_*) while utilizing the reduced features (Phase 3 of Figure 2) for crowd condition prediction using the feature selection method (*CCPFSM*) to enhance the proposed approach shown in Equation (5) in Phase 4 of Figure 2 using Equation (3). The *EHCAF* is discussed as follows:

*EHCAF* = *IAR_ehcaf_* + *IBE_ehcaf_* + *CCPFSM* (3)

where *EHCAF* comprises the improved *SBTFD* and reduced features from the FSM in our previous work [8]. *IBE_ehcaf_* represents the newly reduced features achieved using the employed FSM combined with Vsi and Dsi performed for IBE implementation with the modified and adopted algorithms (1) and (2). This serves as input to the modified Algorithm (3) in Figure 2, and are employed in this article. Note that the detail about improved SBTFD features and dimensionality reduction based on FSM (phases 1–3) are out of the scope of this article. 

*CCPFSM* denotes the prediction achieved by the reduced features and other parameters known as flow velocity V_si_ and flow direction D_si_ in Equation (2) (Phase 4), which were used to perform a task for the prediction of crowd condition in Equation (3). It employs an enhanced context-aware framework through the use of context-sensing from node S and crowd density determination (CDD) in Phase 4 for the inflow and outflow movement of individual behavior to evaluate the possible causes of abnormality in a crowd using the proposed approach as a solution. This helps to realize the development of *EHCAF* shown in Equation (3). 

#### 3.2.2. Modified Algorithm for Region Identification and Grouping of Nodes S 

Crowd behavior monitoring was done with the use of sensor signals for identifying each participant with a smartphone as node S, based on an individual followed up by a grouping of the nodes (S) (see Algorithm 1 in Appendix A). It was conducted using the individual sensor analyses in Step 4 (Section 3.1) with context recognition performed on the activity recognition of an individual, in order to estimate participants’ behavior. The mapping between the program sensors and activities considered were utilized as input to algorithm 1 (Appendix A) implementation. In Algorithm 1, S is the participant node used as input in Step 4 (Section 3.1). 

The crowd formation distribution is divided into sets of sub-regions using the crowd controller station (CCS). When a new participant node S is detected, the context-aware application notifies the crowd controller station, which automatically adds the new node to the specific sub-region of the present location in line 19 (Algorithm 1 in Appendix A). The region identification of participant is actualized with the smartphone of the participant as a node S, line 1, with the GPS data in lines 2–3 with respect to time (line 4 of Algorithm 1 in Appendix A) using the data displayed in Figure 1. 

The grouping of participants into the sub-region list SA_1_, SA_2_, and SA_n_ is achieved using line 20 of Algorithm 1 in Appendix A. It takes care of the movement of the participant from one place to another for the scenario used in the experiment. Node S was equipped with the context-aware mobile application prototype during the experiment, whenever the distance moved by the participant is greater than a threshold value in (line 18 of Algorithm 1 in Appendix A), as adopted in the work of [6]. The threshold value is about 20 m from the hotspot for effective monitoring via communication within the coverage area. Once the node is outside the hotspot range, it is exempted. The algorithm also determines the neighbouring nodes in a sub-area by estimating the distance between two participant nodes and other nodes monitored by the CCS. Based on the work of [6], if the distance between nodes is less than 10 m, the new participant node will be added to the same area using line 19 of Algorithm 1 in Appendix A. The distance of 10 m was selected for the hotspot to allow for ease of assessments in case of an emergency. The distance estimation is based on Vincenty’s formula and is adopted for computing latitude and longitude coordinate points [5,44].

#### 3.2.3. Flow Velocity Estimation and Flow Direction Identification Based on Activity Recognition

The implementation of this algorithm takes the contexts from sensor signals—specifically latitude, longitude (GPS data), accelerometer x, accelerometer y, accelerometer z, and timestamp—as input to Equation (3) of Figure 1. The input data were used to compute the flow velocity estimation and also used to determine the flow direction of individual movement behavior. The output from the implementation of the algorithm is flow Velocity (*V_si_*) and flow Direction (*D_si_*) [44]. The *V_si_* and *D_si_* are important informative features used to obtain hidden context information from individual behaviors in a crowd scenario that is considered to determine flow patterns of individual movement. 

#### 3.2.4. Implementation of Modified PBEA Algorithm 

The disparity matrix is the difference between a node and any other nodes used in (Algorithm 2 of Appendix B). For example, u and v; *s_i_* or *s_j_*. The diagonal elements of the disparity matrix are usually defined as zero, which implies that zero is the measure of disparity between an element and itself [44,45]. 

Given two R-dimensional $x_{i}=(x_{i}^{1},x_{i}^{2},\ldots x_{i}^{R})$ and $x_{j}=(x_{j}^{1},x_{j}^{2},\ldots x_{j}^{R}),$ the Euclidean distance (EUD) *d (i, j)* as observed in [45] is expressed in Equation (4):

(4) $$d_{i,j}\;\sqrt{{\left(x_{i}^{1}-x_{j}^{1}\right)}^{2}+{\left(x_{i}^{2}-x_{j}^{2}\right)}^{2}+\ldots +{\left(x_{i}^{R}-x_{j}^{R}\right)}^{2}}$$

where *d i,j* denotes the Euclidean distance in Equation (4).

The computation was performed to calculate the distance between nodes for the input data from S1 to S20. This is to determine the disparity value for individual estimation in each region where node S is located. The variables $x_{i}^{1},x_{j}^{1}$ correspond to the features and their instances in pairs; based on SBTFD, a reduced feature set (fft_corxz, y_fft_mean, z_fft_mean, z_fft_min, y_fft_min, z_fft_std, y_fft_std) is then combined with Vsi and Dsi contexts from the sensor signals of D1. These serve as input to the PBEA. Euclidean distance (EUD) is commonly used in research across different domains. It has been used to compute the distance between two points with reliable results; hence, the choice of using it to generate distance from each participant to every other participant based on nodes [45,46]. In addition, the investigation revealed that EUD is suitable for the modified PBEA adopted from the BCF implemented in this research.

The algorithm caters for n numbers of nodes, but the location used for an experiment does not vary for all the activities performed. This was due to the aforementioned communication range stated in (Algorithm 1 of Appendix A). Thereafter, the clustered results obtained were similar beyond three sub-areas, since the location considered is uniform for the experiment. This was noticed from the GPS data for longitude and latitude obtained in the experiment used with D1. It was observed that there is a variation between nodes whose monitor’s device is represented by S for identification. The cluster of nodes was performed using Equation (5):

(5) $$EUD\;(d_{i,j})=\sum_{i=1}^{n}\sum_{p\;\varepsilon \;K_{i}}dist{\left(p,k_{i}\right)}^{2}$$

In Equation (5), EUD represents the Sum of the Square Error (SSE). SSE is determined by using the node of the participant that is nearest to each pair of the participant node, which helps for S identification in the monitoring group and subsequent ones in the group. The advantages of K-means that were adopted and used in Algorithm 1 in Appendix A were discussed in [44,46]. Equation (6) was applied to perform the IBE*ehcaf* in Equation (3) (of Phase 4). 

For the IBE_ehcaf_ task, let *δ* be a matrix of pairwise between *n* attributes in Equation (6) [26]:

(6) $$\delta_{i,j}\;=\left(\begin{array}{l} \delta_{1,1}\;\;\delta_{1,2}\;\;\delta_{1,3}\;\;\delta_{1,4}\ldots \delta_{1,n}\\ \delta_{2,1}\;\;\delta_{2,2}\;\;\delta_{2,3}\;\;\delta_{2,4}\ldots \delta_{2,n}\\ \delta_{3,1}\;\;\delta_{3,2}\;\;\delta_{3,3}\;\;\delta_{3,4}\ldots \delta_{3,n}\\ \delta_{n,1}\;\;\delta_{n,2}\;\;\delta_{n,3}\;\;\delta_{n,4}\ldots \delta_{n,n} \end{array}\right)$$

where $\delta_{i,j}$ represents the disparity between the aforementioned features *i* and *j*. Also, let *f (*$\delta_{i,j}$) be a monotonically increasing function that transforms differences into disparities using Equation (6).

The equation produces an R-dimensional matrix (where R ≤ n) configuration of points. 

$x_{i}=(x_{1},x_{2},\ldots ,x_{i},\ldots x_{j},\ldots x_{n})$; likewise, $x_{i}=(x_{i}^{1},x_{i}^{2},\ldots x_{i}^{R})$ and $x_{j}=(x_{j}^{1},x_{j}^{2},\ldots x_{j}^{R}),$ for (*1 ≤ i, j ≤ n*). The *EUD* between any two nodes, S of $x_{i}$ and $x_{j}$ in this configuration, equals the disparities between features *i* and *j* expressed using Equation (7):

(7) $$d_{i,j}\approx f(\delta_{i,j})$$

The $d_{i,j}$ is defined by Equation (6). The measure has been applied to find the pairwise (Euclidean distance) between two cities with minimum possible distortion by [47], as reported in [46]. In this case, we represent the *n* nodes of the matrix D (N, A) where u =N and v =A for B^(s)^ with the positive integers 1, 2, 3,... n. Then, a distance matrix, B^(s+1)^, is set up with elements, and is expressed using Equation (8) [46]:

(8) $$d_{0}(i,j)=\left\{ \begin{array}{cc} l(i,j) & if\;participant(node)(i,j)\;exist\\ d_{i,j}=0 & if\;i=\;j\\ d_{i,j}>0 & if\;i\;\neq \;j \end{array}\right.$$

The length, *d(i, j)*, of the path from node *i* to node *j* is given by element D (*u*, *v*) of the final matrix D^(n)^ B^(n)^, which makes it possible for the tracing back of each one of the node paths. An example of disparity matrix computation can be computed using Equation (9) as employed for the participant estimation algorithm noted in [5,24]:

(9) $$D_{\left(u;vT\right)}\;=g(Corr(f(B_{si},T),\;f(B_{si\;+\;1},T)))$$

where D is the disparity based on function *f*, and g is a variable that provides the mapping to a disparity value *f*. The disparity value is computed based on the input data, specifically fft_corxz, y_fft_mean, z_fft_mean, z_fft_min, y_fft_min, z_fft_std, y_fft_std, *V_si_* and *D_si_*. While *f* depicts correlation (*Corr*) performed on a matrix containing the input data in pairs; *B_si_* is an individual participant node; *u* is the number of nodes of the participant along the column of the matrix; *v* is nodes of the participant along a row of the matrix, and *T* denotes time. The function *f*_,_ Corr, and *g* depend on the specific crowd that is considered. Typically, *f* is a pre-processing function. Corr computes a measure of differences between the input data for every (*i, j*) pair of nodes to determine an individual in a crowd scenario. Finally, g maps to a disparity value. The disparity value is defined to be zero if the two participants are likely resulting from their participation in the same crowd. Conversely, the disparity tends to one or more if the node *s* is not likely to be the result of participation in the same crowd. The outcome generates a disparity matrix $D_{T}={\left[D_{\left((u;vT\right)}\right]}_{m\;\;x\;n}$ at time *T*. The reduced features set achieved and other parameters derived as features previously reported in [33]—namely, *V_si_* and *D_si_* [44], are fed into the PBEA, as shown in Equation (6) of (Phase 4) as input to generate the output for individual and crowd condition prediction illustrated in the next section. 

#### 3.2.5. Crowd Density Threshold Condition

This study adopted the conditions that trigger abnormality to set a threshold for crowd density determination within the coverage area as established in previous studies [48] and employed in other studies [3,4,6,49]. The threshold adopted in this study was first suggested by [6], who defined a crowd as made up of three or more persons. This study employs two persons per m^2^ for the experiment based on [6]. However, the monitoring of participants occurs within the coverage areas and range of distance for the hotspot, and can be assessed using the device of a participant smartphone, which is referred to as node S. It is generally acknowledged that five persons/m^2^ is an extremely high density, four persons/m^2^ is high density, three persons/m^2^ is medium density, two persons/m^2^ is low density, while one or no persons/m^2^ is considered very low density [7]. In addition, six or more persons/m^2^ is considered extremely dangerous, with the potential to cause abnormality [7]. Crowd density determination (CDD) was employed to compute the density of the monitored crowd of moving nodes based on a crowd density threshold (CDT) condition shown in Equations (10)–(12) of (Phase 4). Node S is recognized by the crowd controller station (CCS) based on node count using Equations (10) and (11) [50].

(10) $$Density\;=\;LN\;<\;area\;in\;m^{2}\;*\;5$$

(11) $$\mathrm{CDD}=1+4*\left[\frac{Density-\lambda }{\psi -\lambda }\right]$$

where *LN* represents the number of participants monitored, λ denotes the minimum density level, and ψ is the maximum density observed in the experiment at a particular time. The maximum capacity has also been proposed to be calculated using the number of participants < area in m^2^ × 10; where 10 is regarded as extreme crowd density, as noted in the work of [50]. More than two participants per m^2^ exceed the threshold. In order to explain the disparity matrix (a low value and high value) employed by [5], which is used to explain the type of crowd observed in the analyses of the result for this article, Equation (12) shows the crowd density threshold condition (CDT) used for the CDD evaluation.

(12) $$\begin{array}{l} \left\{\begin{array}{c} 1.\;If\;CDT\;for\;d_{i,j}\;per\;sqm^{2}\leq \;2\;then\\ low\;crowd\;density\;occur\\ 2.\;else\;If\;CDT\;for\;d_{i,j}\;per\;sqm^{2}=\;3\;then\\ medium\;crowd\;density\;occur\\ 3.\;else\;If\;CDT\;for\;d_{i,j}\;per\;sqm^{2}=\;4\;then\;\\ high\;crowd\;density\;occur\\ 4.\;else\\ extremely\;high\;crowd\;density\;occur \end{array}\right\} \end{array}$$

## 4. Experimental Results 

This section presents results based on the highlighted objectives as follows: the raw sensor data validation, and the descriptive analysis for the validation summarized for all classes N: 22,350, which consists of V1 to V8. V12 provided a mean of 4.735, the standard deviation of 2.519, and a standard error of 0.2216. V13 provided a mean of 47.762, the standard deviation of 47.501, and a standard error of 0.4179. V15 produced a mean of 21.629, the standard deviation of 82.162, and a standard error of 0.7228. Meanwhile, V18 provided a mean of 48.891, the standard deviation of 106.286, and a standard error of 2.255. Inferential statistics for the ANOVA test conducted at *p* = 0.05 shows V12, V13, V15, and V18 having F-values of 46644.20, 4653.71, 196.41, and 967.01, respectively. The *p*-value = 0.000 is statistically significant. Hence, we reject H_0_, and accept H_A,_ and conclude that there is a significant difference in at least one of the means of the variables V12, V13, …, V18 used for the analysis of D1. This conclusion implies that the D1 dataset is valid, consistent, and adequate for the analysis conducted in this study. 

### 4.1. Result on the Classification of Raw Dataset D1 

**Figure 3:**
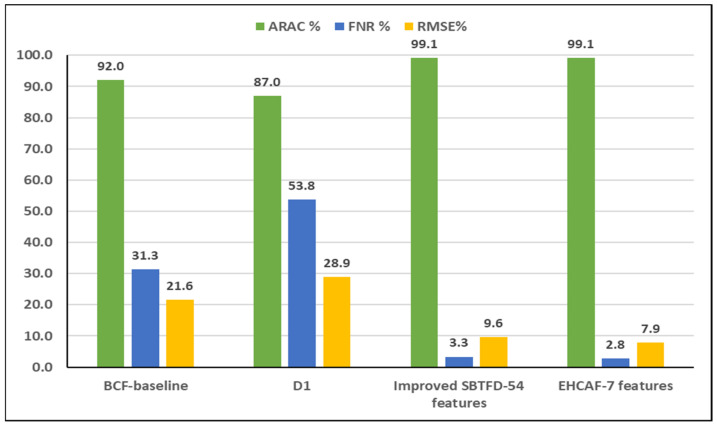
Comparison of BCF—baseline classification results, raw dataset—D1, improved statistical-based time-frequency domain (SBTFD), and reduced features for the enhanced approach.

The results of classification after validation is as follows. In Table 5, out of the 22,350 instances (last row); about 10,692 (bold in diagonal) of the confusion matrix were correctly predicted, while the remaining 11,658 instances were wrongly predicted. In Figure 3, the summary of classification results for baseline, a raw dataset D1, an improved SBTFD with 54 features, and seven reduced SBTFD features newly introduced to extend the BCF to produce an enhanced approach (EHCAF) is presented in Equation (3). The best ARAC, FNR, and RMSE are achieved with EHCAF-7 features having 99.1%, 2.8%, and 7.9%, respectively. This is against 92.0%, 31.3%, and 21.6%, respectively.

### 4.2. Results of Region Identification and Grouping of Nodes Using Clusters

**Figure 4:**
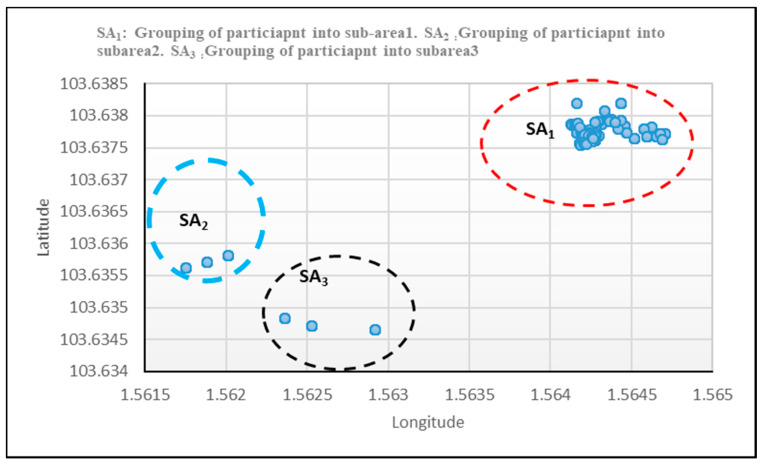
Results of clusters for identifying and grouping participant into subareas with GPS data.

Figure 4 provided a higher number of clusters, which shows that more participant nodes gathered in subarea SA_1_ than subareas SA_2_ and SA_3_ in the experiment. Thus, SA_1_ is more prone to risk than SA_2_ and SA_3._

### 4.3. Results on the Algorithm Implemented for Flow Velocity and Flow Direction

For details of the algorithm implemented for flow velocity and flow direction, please refer to [44]. This article focuses on the individual behavior estimation method combined with reduced features, which were not considered in the BCF. 

### 4.4. Modified PBEA Using Reduced Features and Enhanced Individual Behavior Estimation

The output serves as input to the modified PBEA as shown in Figure 2 to produce an enhanced context-aware framework for individual and crowd conditions. The analysis is based on pairs of the node; for example, 1 and 2, 1 and 3, 1 and 4... up to 20 for individual behavior estimations. A disparity matrix was computed for the estimation of an individual based on the 20 nodes used as input for S1 to S20 for different nodes in the experiment. The experimental result revealed the interaction of participating (nodes) and their behavioral patterns in a crowd scenario based on the CDT employed and crowd density estimate. It shows two, three, three, and 12 nodes of a different number of individuals per m^2^ (Appendix C).

#### 4.4.1. Crowd Condition Prediction Using Individual Behaviour Estimation

For crowd estimation, it is necessary to estimate individual activity recognition and behavior initially. This had been addressed in our earlier works [4,8]. The crowd condition prediction using seven reduced features with Vsi and Dsi is newly introduced. This achieved higher accuracy by 99.1% against 92.0%. Also, a marginal reduction of the false negative rate by 28.5% from 2.8% against 31.3%, which is an improvement over the BCF [5], was obtained to achieved EHCAF see Figure A2 of Appendix D. The individual behavior estimation with suggested CDT and crowd density determination computation for crowd count serve as a means to extend the BCF [5]. This could help identify early danger by using context sensing through a smartphone with a context-awareness alert, thus minimizing the level of abnormality behavior in a crowd-prone area. 

#### 4.4.2. Implication of Low False Negative Alarm on the Enhanced Approach Based on PBEA Experiment

**Figure 5:**
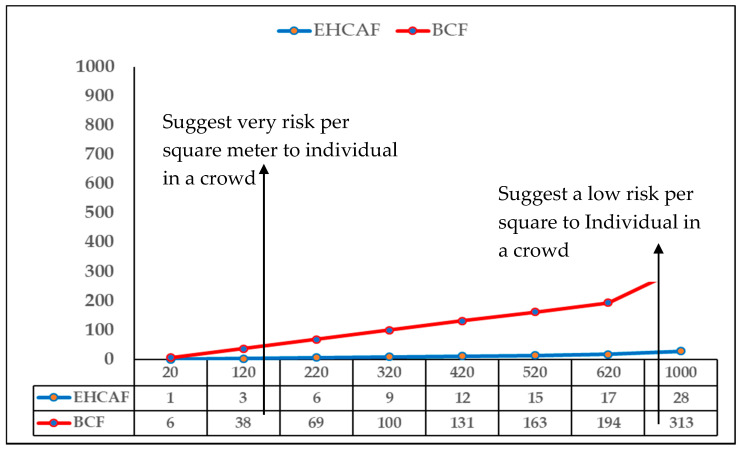
Effects of the false negative rate on the proposed approach when applying to human behavior monitoring in real life in a crowd condition.

Figure 5 shows that the experimental results based on the proposed approach using reduced features and enhanced IBE in this article for crowd condition prediction has a low false negative rate (FNR), achieving an FNR of 2.8% and an ARAC of 99.1%, compared with an FNR of 31.3% based on an ARAC of 92% in the baseline. The results suggest that the higher the false negative rate (FNR) of AR, the higher the number of participants that may be at risk. Figure 5 also shows the comparative risk situation for EHCAF in blue color and BCF in red color, showing one (1) participant (node) in 20 and 28 participants in 1000 for the EHCAF, and six in 20 and 313 participants for 1000 in the BCF. The value was computed using a FNR of 2.8/100 * Number of the participants (NOPs) based on a crowd of people considered which will be varied in a real-life scenario when the proposed is applied.

This section presents the details of benchmarking with related works in the literature [5,51,52]. To confirm that the achieved higher results for the proposed approach is significantly better on the evaluation measurements used, Statistical t-tests were carried out using SPSS version 22.0 on dataset D1 and the BCF. The results of the seven reduced features based on FSM from method A, with *p*-values of 0.003 for the improved SBTFD and 0.021 against BCF, indicates *p* < 0.05, implying that the performance of the proposed approach is statistically significant at an 0.05 alpha level. 

This supports the objective presented in this article. Based on the analysis of results, the enhanced context-aware framework (EHCAF) depicted in Figure A2 (Appendix D) is an improvement on the basic context-aware framework (BCF) benchmark, as shown in Table 6. However, Table 6 shows the components for EHCAF; likewise, the justification for improved parameters to establish the validity of our findings in the entire study.

## 5. Discussion of Results

**Figure A1:**
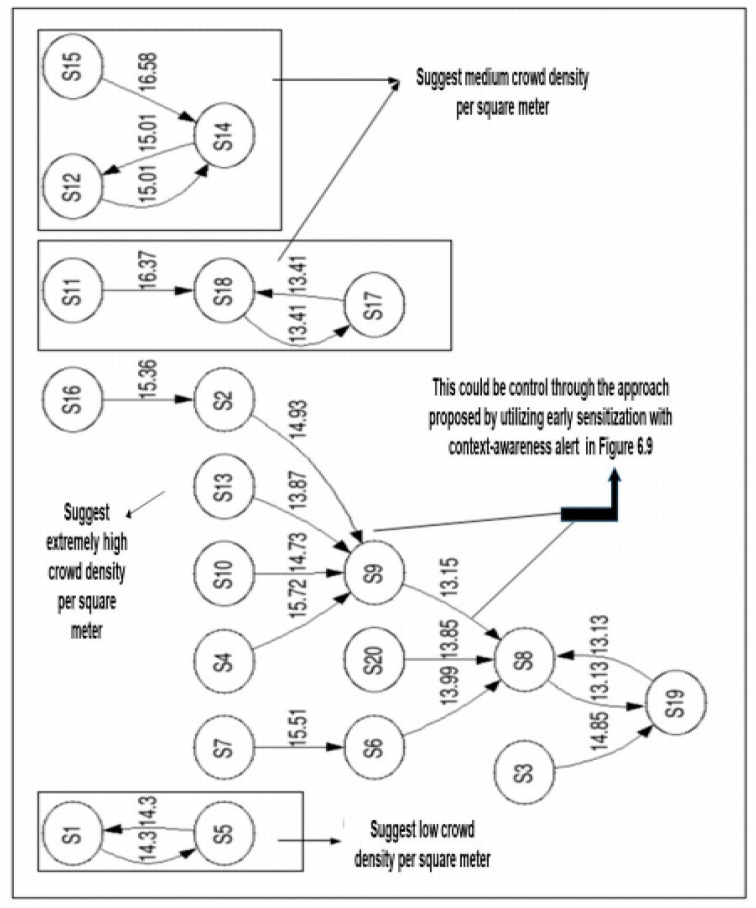
Patterns of participant behavior estimation using a disparity matrix for 20 nodes, S1–S20, for the recognition of abnormality of individual behavior per m^2^.

The result achieved an improvement of 7.1% and a false negative rate of 28.5% with an error reduction of 13.7% in terms of root mean square errors. This suggests safety to human lives in a crowd-prone situation when applying to real-life applications against the BCF by [5] as analysed in Table 7. In Figure 4, the susceptible area where crowd abnormality is likely to occur suggests sub-area list *SA*_1_; this was obvious from the plot as more clustered nodes were observed in the area, which is an indication of more participants interacting together at a very close range to one another, as shown in Figure A1 (of Appendix C).

Based on the flow velocity Vsi and flow direction Dsi from accelerometer sensor signals analyzed, the V3 fall scenario revealed that only 778 were correctly recognized as TP, out of the 3159 expected among the instances of 22,350. Meanwhile, the rest consists of FP: 2383, FN: 2831, and TN: 16808 in Table 5. In Table 5, the unrecognized individual activity from 2381 which accounted for the abnormal behavior of individuals could be responsible for disaster manifestation. In a nutshell, the incorrect recognition demands effective features such as those suggested with the statistical-based time-domain in [10,11,12,13,14,15,16] and statistical-based frequency domain in [27,52], which informed the solution adopted in our previous work [4,33]. 

**Figure A2:**
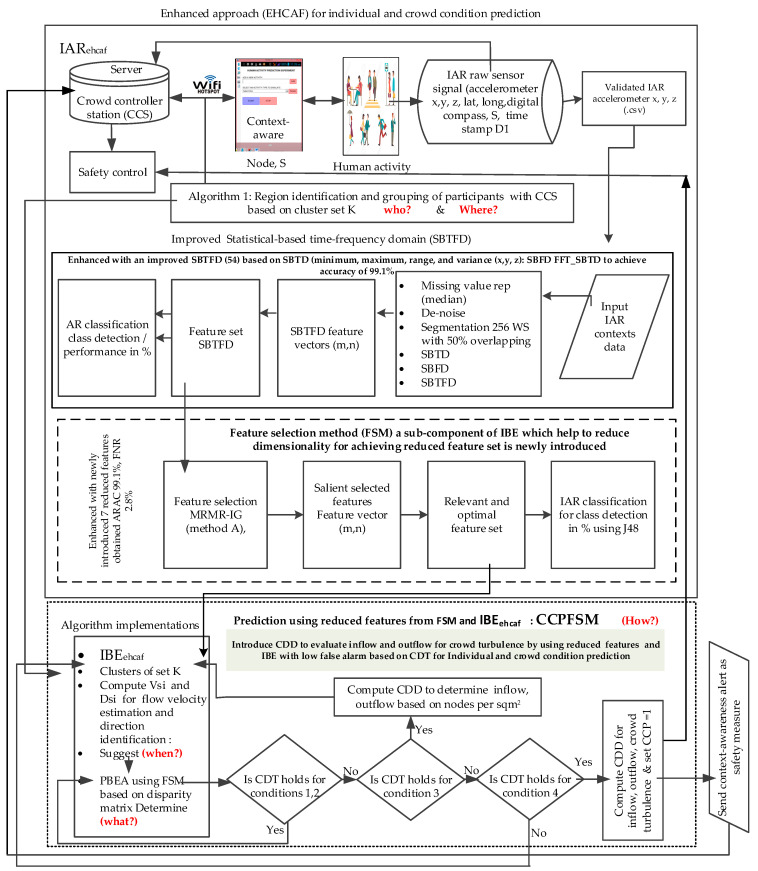
Patterns of participant behavior estimation using a disparity matrix for 20 nodes S1 to S20 for the recognition of abnormality of individual behavior per m^2^.

Figure A1 (Appendix C) showed four distinct groups with the highest and lowest number of participants with 12, three, three, and two nodes, respectively. It shows the interactions and range at which those nodes interconnected for the scenario used as an example. Another plot from the data using a different set of 20 nodes to compute a different set of disparity values based on the disparity matrix with implemented algorithm three gave a similar result. The 12 nodes suggested a dangerous situation in terms of crowd scenario according to [6,7]. This implies a high inflow and outflow, which could bring about high crowd turbulence, and thus requires an immediate control if it happens in a crowded situation. All three nodes in Figure A1 (Appendix C) signify a medium crowd density, and the two nodes indicated a very low crowd density, which is basically known as a normal situation. Therefore, it is found to be within the threshold suggested using Equation (11). Based on this, the pattern of 12 nodes using an undirected graph in real life may result in crowd abnormality occurrence. In such cases of the 12 nodes with early recognition and sensitization using the proposed context-aware framework, such crowd density can easily be controlled before it reaches a critical state. Most importantly, for example, in Appendix D, with an FNR of 2.8% for every 20 and 1000 participants (nodes), which were assumed to be monitored one node and 28 nodes, respectively, will be at risk using the proposed solution, versus six and 313 nodes respectively in the basic context-aware framework (BCF) [5]. Experimental results support activity recognition studies in the literature for both cross-validation and split [11,39]. It also identifies that RF and J48 are the best classifiers suitable for the enhanced context-aware framework (EHCAF) Figure A2 Appendix D for individual and crowd condition prediction as compared to the other classifiers investigated. In view of our findings, the limitation of this work includes an inability to develop a context-aware system to effectively implement the reduced features that are newly suggested in this research. Future work could investigate and integrate the use of this methodology to the realization of safety for human lives through viable application in real life. Also, there was an inability to handle the technicality on the part of the monitoring device functionality to identify none of the functional sensors that could hinder the smooth data acquisition of individual activity recognition for prediction. 

## 6. Conclusions

This study has described the sensor signals of activity recognition that are adequate for the prediction of individual and crowd conditions. The entire approach demonstrated in this article fulfills the aim, which focused on complementing other research in human activity recognition and pervasive computing toward the mitigation of crowd abnormality in the 21st century. In this article, an enhanced context-aware framework (EHCAF) was developed. The potential of reduced features with the feature selection method based on the improved feature extraction method using SBTFD was demonstrated. The relevant parameters were derived and applied to implement the modified algorithm for grouping participants using smartphones as nodes. Based on findings, an enhanced approach for individual and crowd condition prediction is summarized as follows: the utilization of reduced features and enhanced individual behavior estimation (IBE_enhcaf_) with high accuracy and low FNR performance is achieved; a clear definition of crowd density formulation for crowd condition prediction in a crowd scenario is presented. Above all, from the previous study, the FNR is 31.3%, while in this study, it is 2.8%. Hence, an improvement of 28.5% is achieved based on the experiment. However, the limitations and gaps left by previous studies have been equally addressed. The experimental results of this article have shown significant improvement from the previous studies done by [5,11,24,39]. The methods applied to achieve the proposed enhanced approach showcased in this article support the objective of the article. In the future, the approach promises a dynamic solution that intends to explore the collection of the ground truth dataset for the purpose of mitigating disasters among individuals gathering in places such as Mecca, medina during the pilgrimage in Saudi Arabia by integrating cloud-based technology. 

## Figures and Tables

**Table 1:** Strength and limitations of existing feature extraction methods.

Feature Domain	Feature Extraction Methods	Merits	Demerits
TD	Mean	Is a good discriminator of individual characteristics calculated with small computational cost and a small memory requirement, is commonly used a feature in activity recognition (AR) research [12,16,22]	Does not produce a good result when isolated from other measures.
	Standard deviation	Derived through the use of mean to reveal any deviation in AR sensor data [6]	Frequency domain absence hinders its performance
	Correlation	Help to determine the correlation between one individual’s characteristic feature and the other to express [6].	Failure to produce the FD along the corresponding axis affects the performance of AR accuracy.
	Root Mean Square	Quality of sensor’s data may dictate its tendency to reveal the actual location for individual in the prediction of crowd disaster [6].	Could not work in isolation from other measures.
FD	FFT_RMS	Good tool for stationary signal processing [6,18].	Weakness in analysing non-stationary signals from sensor data.
TDFD	Time domain -frequency domain	Produce an efficient performance for individual’s representation in the crowd [6,14].	The use of FFT_RMS as the only FD may not assume the performance of other TD features.

^1^ Note: TD = Time domain feature; FD = Frequency domain feature; TDFD = Time domain–frequency domain feature; FFT_RMS = Fast Fourier Transform of Root Mean Square.

**Table 2:** Summary feature extraction methods (FEM) methods used and those that have not been used in crowd-related studies.

Feature Extracted Methods in Activity Recognition	Application Domain	Features That Have Been Used in a Crowd	Reference
DD: Discrete cosine transform (DCT) 48 coefficients DCT features extracted	Daily activity	N/A	[25]
Variance (Var.) ax, ay, and az; number is not specified	Crowd behavior	Var. along x, y, and z	[26]
TD: Mean; std.; mad; max; min; sma; interquartile range (Iqr); entropy; arCoeff; cor.; maxfreq.; meanfreq.; FD: Max; min; sma; interquartile (iqr); skewness; kurtosis, energy band; angle; TDFD: 561 features	Daily living activity	Mean, Std, along x, y, and z	[18]
TD: mean, std., correlation (corr.), rms a_x_ a_y_ a_z_. FD: FFT_rms a_x_ a_y_ a_z_; TDFD: 15 features	Crowd abnormality monitor (CAM)	Features in the baseline study Known as BCF	[6]
TD: all time domain features in Table 1; FD: spectral coefficient; max. frequency; entropy of coefficient; dominating frequency; discrete coefficient; empirical cumulative distribution function (ECDF): with the setting of parameter value based on bin used for inverse computation; number is not specified	Motion sensing in daily life	Mean, Std, along x, y, and z	[27]
TD: mean, max, min, std., zero cross, median, range, sum of square, rms and var. TD: 30 features	Individual activity contexts	Mean, Std, along x, y, and z	[11]
TD: Mean; std.; max.; min.; corr.; Iqr.; DD: Dynamic time warping distance discrete time wavelet (DTW). FD: FFT coefficients as frequency domain features; except the first FFT coefficient. WD: wavelet energy TDFD and WD: 89 features	Motion sensor for daily activity	Mean, Std, along x, y, and z	[28]
TD: min, max, mean, STD, signal magnitude area (SMA), signal vector magnitude (SVM), tilt angle, FD: power spectral density (PSD), signal entropy, special energy: 60 features	User’s daily detection of abnormality	Mean, Std, along x, y, and z	[29]
Improved SBTFD features presented in our previous work	Individual and crowd condition prediction	15 features are newly suggested as improved TD for SBTD, and 24 features as improved FD for SBFD	[4]

**Table 3:** Related context-aware frameworks and activity recognition methods with the research gaps for individual and crowd condition prediction.

Context-Aware Framework/AR	ARAC	FSM	CCP	Features Used	Why the Features Are Not Enough
CAM-BCF [6,42]	92% based on TDFD	N/A	A high false negative rate	TD: mean x, y, z, std. x, y, z; cor. xy, yz, xz; rms. x, y, z; FD: FFT rms along x, y, z-axes as TDFD features	Salient TD and FD features with better result commonly used in literature were overlooked
IDAS [36]	N/A	N/A	N/A	N/A	N/A
Context recognition [11]	55–98% based on TD	N/A	N/A	TD: Mean, STD.; Med. Min., Max., Zero Crossing, (ZC), Sum of Squares (SOS), rms, Range, Var	Attention paid to the only TD without giving consideration to FD that compliments TD features
Feature analysis [42]	86–93% based on FSM	CFS, CHI, MRMR	N/A	75th Percentile (PE): PE_y, min-max: mm_x, mm_y, PE_x, mm_z, PE_z	Negligence of FD features in selected features and 86.6% reported for MRMR
Coupling HAR [43]	86–91% based on TDFD	N/A	N/A	Not specified	The detail was not given

^3^ Note: ARAC = Activity recognition accuracy, AR = Activity recognition, FSM = Feature selection method adopted to reduce features and CCP = Crowd condition prediction. CFS = Correlation-based feature selection, CHI = Chi-square feature selection and MRMR = Minimum redundancy–maximum relevance feature selection.

**Table 4:** Summary of sensor signals for the D1 raw dataset based on experiment conducted.

Attribute	Dataset 1 (D1) [4]	Class	Activity/Sensors Name
Age	25–51 years	V1	Climb down
Activity count	8	V2	Climb up
No of instances	22,350	V3	Fall
No of participants	20	V4	Jogging
Sensor type	Accelerometer x, y, and z digital compass (DC), longitude, latitude, timestamp	V5	Peak shake while standing
Position placement	Hand	V6	Standing
No. of devices	20 smartphone	V7	Still
Dataset gathering	Crowd controller as a server	V8	Walking
		V12	Latitude
		V13	Longitude
		V14	Speed
		V15	Altitude
		V16	Timestamp
		V17	Digital compass
		V18	Accuracy

**Table 5:** Confusion matrix from the classification result of individual activity recognition (IAR) using the sensor signals of the D1 raw dataset.

Class Label	Predicted Class								Actual Class
	V1	V2	V3	V4	V5	V6	V7	V8	TP + FN
**Climb down: V1**	**591**	425	228	147	106	137	41	300	1975
**Climb up: V2**	405	**705**	292	178	161	186	57	426	2410
**Fall: V3**	188	273	**778**	325	858	254	99	384	3159
**Jogging: V4**	147	163	269	**1698**	190	131	42	312	2952
**Peak shake_wst: V5**	113	161	854	233	**767**	101	24	144	2397
**Standing: V6**	106	142	210	110	70	**1813**	85	221	2757
**Still: V7**	40	67	112	49	47	110	**2733**	72	3230
**Walking: V8**	273	380	418	312	159	255	66	**1607**	3470
**Total**									**22350**

**Table 6:** Comparison between BCF [6] and proposed approach (EHCAF).

Components	EHCAF	Justification
	IAR_ehcaf_	
AR dataset	Validation of D1 performed with ANOVA is significant	Explain the suitability of the D1 in line with the literature. Quality of data is very important for crowd monitoring and accurate prediction
Accuracy	99.1%, 98.0%, and 99.0% were achieved	An improvement over BCF with enhanced accuracy performance is achieved
Feature selection method (FSM)	Minimum Redundancy Maximum Relevance with Information Gain (MRMR-IG) with SBTFD provided seven reduced features (Corr_xz-fft, y_fft_mean, z_fft_mean, z_fft_min, y_fft_min, z_fft_std, and y_fft_std)	Reduces the dimensionality of features space on the monitoring devices. Lower computational task. Facilitates early recognition and utilizes less time for classification
Classifier	J48, Random forest (RF)	Compatible with an Android device and widely used in AR
Accuracy & FNR	99.1%; 2.8%	Improvement of 7.1% accuracy and 28.5% FNR over BCF
Individual Behavior Estimation	IBE_ehcaf_	Provide accurate prediction to enhanced the safety of human lives
Region identification	Modified algorithm using k-means to implement Algorithms 1 and 2 with D1 to identify the region, cluster nodes S, and group into sub-areas	Potential to reveal susceptible clusters nodes in sub-areas that are prone to danger. Ascertain threshold with the specify coverage of nodes
Grouping of node S into Sub-area		
Flow velocity and flow direction	Adopted and implemented using D1	Serve as informative features to extract individual context behavior not possible for IAR in phases 1 to 3
IBE	Modified PBEA using flow velocity (Vsi), flow direction (Dsi), and seven reduced features for IBE	Estimation of nodes per m^2^ and analysis within coverage areas experimented with volunteers
Threshold	Threshold > two per m^2^	An efficient method should measure accurately the number of volunteers (node) within per m^2^ to prevent abnormality occurrence in a crowd.
Inflow, outflow & crowd turbulence	Compute and evaluated using CDD based on individual count	Potential to identify person prone to danger early using context-awareness alert
Crowd condition	Crowd abnormality behavior	To enhanced the safety of human lives in a crowded area
Prediction	Crowd condition prediction using modified PBEA with reduced features (CCPFSM)	Enhanced approach with improved accuracy and FNR performance
Validation	Inferential statistics and paired sample statistics test was used to validate all the three methods employed for the enhanced approach	Improved SBTFD with 0.002; reduced features with 0.003 and 0.021 of *p* < 0.05 are statistically significant

**Table 7:** Comparison of the proposed approach (EHCAF), activity recognition, and basic context-aware framework (BCF).

Context-Aware Frameworks	SCI	ARAC	FEM	FSM	CCP	RMSE
BCF-baseline [5]	✓	92.0%	TDFD-15	N/A	High FNR (31.3%)	21.6%
[11]	✓	55% to 98.0%	TD-30	N/A	N/A	N/A
[40]	N/A	N/A	TDFD Wavelet	MRMR 86.6%	High FNR (56.5%)	31.0%
Proposed approach (EHCAF)	✓	99.1%	Improved SBTFD-54	7 reduced features using MRMR-IG (method A)-99.1%	Low FNR (2.8%)	7.9%

Note: SCI: Context-aware issues. ARAC: Activity recognition accuracy. FEM: Feature extraction method. FSM: Reduced features achieved using Feature Selection Method. CCP: Crowd Condition Prediction. RMSE: Root mean square error. N/A: Not applicable.

## Appendix A

**Algorithm A1.** Modified algorithm for region identification and grouping of participants based on clusters using K-means with node S
1. **Set** S: node for participant’s smartphone
2. **Set** Lat: Latitude
3. **Set** Long: Longitude
4. **Set** T: Time
5. **Set** SA: Sub-arealist = [SA_1_, SA_2_, SA_3_,…, SA_n_]
6. **Set** Dist: Distance
7. **K**: Clusters of nodes into sub-areas
8. **TWindow**: Time T, set for the location of nodes a threshold
9. **Start**
10. **Input** S: Output (Lat, Long, Time)
11. **Input** Sub-area list [SA_1_, SA_2_, SA_3_,…, SA_n_, Lat, long, T]
12. **Output** S clusters in Sub-areas, SA_n_
13. **While** S is ready **do**
14. **For each** S **for participant in Sub-Arealist do**
15. Set locationUpdateWindow
16. Set minT i.e., for location manager minimum power consumption with minT
Milliseconds between location update to reserve power
17. Set minDist: as location transmission in case device moves using minDistance
meters
18. TDifference = location.getT( )- currentbestlocation.getT( )
**If** TDifference > TWindow then participant (node) have moved and transmit
the new location into a Crowd Controller Station (CCS) based on timestamp
change
19. **If** (Lat, Long) in location context with Sub-arealist SA_n_ are the same,
clusters set K using Dist between the nodes S
20. Group S into SA_1_, SA_2_, SA_3_,…, SA_n_ clusters
21. Crowdcount = S + 1
22. **End If**
23. **End If**
24. **End For**
25. **End While**
26. **End**

## Appendix B

**Algorithm A2:** Enhanced approach for individual and crowd condition prediction proposed to extend BCF
1. **IAR_ehcaf_ Module**
2. ***Set*** S: as node for a participant using a smartphone
3. ***Set*** CCS: crowd controller station: stakeholder as STHD
4. ***Set*** IAR: Individual activity recognition
5. ***Set*** SBTFD: Improved feature extraction method
6. ***Set*** Vsi and Dsi: Flow velocity and flow direction
7. ***Set*** PBE: Pairwise behavior estimation
8. ***Set*** CCP: crowd condition prediction = 0 for all nodes using S
9. ***Set*** CCP as threshold using equation (11)
10. ***Input*** IAR sensor signals dataset D1 from CCS
11. ***Execute*** IAR for *S* using improved SBTFD
12. ***Execute*** dimensionality reduction using reduced features based on FSM
13. **IBE_ehcaf_ Module**
14. ***Cluster*** node *S* using set *K* based on Algorithm 1
15. ***Compute*** *Vsi* and *Dsi* for each *S* based on Section 3.2.3
16. ***Execute*** PBEA using lines 12 and 15 for each class based on Figure 4
17. **CCP Module**
18. ***Compute*** CDD using equations 9 and 10
19. **If** the threshold satisfies condition 1, ***then***
20. ***Terminate*** the PBE testing
21. ***Else***
22. ***If*** the threshold satisfies condition 2, ***then***
23. ***Terminate*** the PBE testing
24. ***Else***
25. ***If*** the threshold satisfies condition 3, ***then***
26. ***Evaluate*** *CDD* inflow, outflow and crowd turbulence
27. ***Else***
28. ***If*** the threshold satisfies condition 4, ***then***
29. ***Evaluate*** line 26 and set CCP = 1
30. (Send context-aware alert to *S* and *STHD* for safety measure)
31. ***Output*** context-aware alert for CCP based on line 29 using EHCAF
32. ***End if***
33. ***Else***
34. ***Execute** line 14 to 31*
35. ***End if***
36. ***End if***
37. ***End if***
38. ***End***
